# Supplementary figures and images for: Sustained Elevated Blood Pressure Accelerates Atherosclerosis Development in a Preclinical Model of Disease
Source: Int J Mol Sci. 2021 Aug 6;22(16):8448. doi: 10.3390/ijms22168448 (PMC8395088; doi:10.3390/ijms22168448)

Supplementary Figure S1

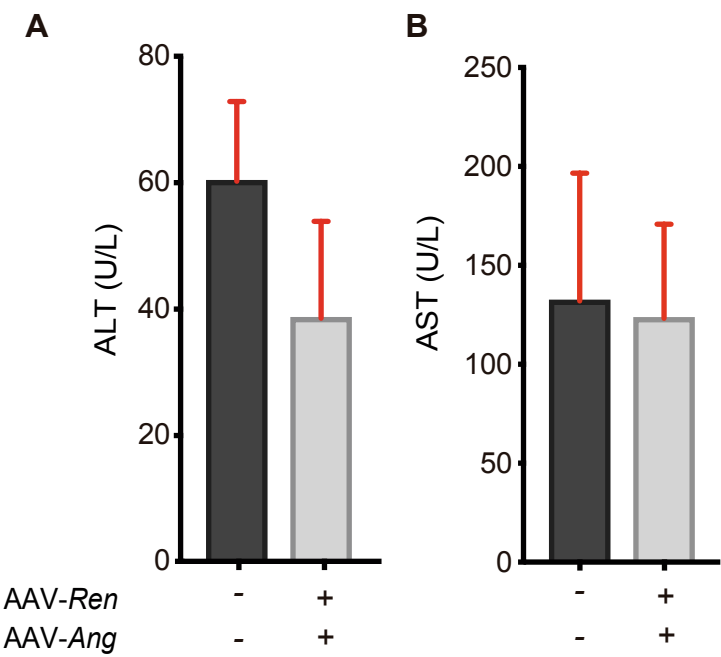

Supplement: Supplementary file 1 [file ijms-22-08448-s001.zip › ijms-1288133-supplementary.pdf]
